# Phenotypic plasticity, trade-offs and gene expression changes accompanying dietary restriction and switches in *Bactrocera dorsalis* (Hendel) (Diptera: Tephritidae)

**DOI:** 10.1038/s41598-017-02106-3

**Published:** 2017-05-16

**Authors:** Er-Hu Chen, Qiu-Li Hou, Dan-Dan Wei, Hong-Bo Jiang, Jin-Jun Wang

**Affiliations:** Key Laboratory of Entomology and Pest Control Engineering, College of Plant Protection, Southwest University, Chongqing, 400715 People’s Republic of China

## Abstract

In this study, we investigated the effects of dietary restriction (DR) and variable diets on phenotypes and gene expression in oriental fruit fly, *Bactrocera dorsalis* (Hendel), one of the most economically important pests in the family Tephritidae around the world. As expected, we found that DR altered the *B. dorsalis* phenotypes by significantly increasing stress resistance and lifespan, but reduced egg production when compared with the control diet. The results suggested a trade-off between reproduction versus somatic maintenance (stress resistance) and lifespan in *B. dorsalis*. Diet also had a significant effect on hatchability, and DR could increase the egg hatching success of *B. dorsalis*. Furthermore, DR up-regulated metabolic pathways involved in energy homeostasis and down-regulated pathways in egg production, which might mediate trade-offs between somatic maintenance and reproduction under DR regimes. The gene expression profiles in response to the acute dietary switches indicated that the digestive and metabolic pathways maybe involved in the adaptability of flies to variable dietary resources. In summary, the research facilitates a better understanding of the molecular mechanisms responsible for the *B. dorsalis*’ phenotypic adjustments to the different qualities of the available diets.

## Introduction

Phenotypic plasticity, the ability of animals to adjust their developmental, physiological or behavioural phenotypes to variable environments, is critical for their survival^1^. The responses of stress resistance, life-history traits and reproduction to dietary quality are important and familiar examples of phenotypic plasticity^2^. Insects obtain energy and nutrients from food; therefore, diet may play a key role in affecting life-history components^3, 4^. Studies of dietary impacts on insects often focus on the morphological and physiological responses of individuals exposed to diets with different qualities. *Drosophila ananassae* has different stress-resistance and life-history strategies, based on the quality of the available diets, that are correlated with phenotypic adjustments at the anatomical level^5, 6^. In tephritid flies, the female reproductive ability can be limited and prevented through denying access to host plants or restricting the dietary precursors of vitellogenesis, i.e., dietary restriction (DR) could extend lifespan and reduce reproduction of Mediterranean fruit fly (*Ceratitis capitata* Wiedmann)^7^. Furthermore, it has also been reported that delaying reproduction of *C. capitate* and melon fly (*Bactrocera cucurbitae* Coquillett) lowers the fitness of females by constraining their fecundity for the remainder of the lifespan without extending the lifespan^8^. Additionally, the previous study also showed that DR in the larval diet had the opposite effect of DR in the adult diet (which prolongs life in this species and across a wide range of taxa) in *Telostylinus angusticollis* ^9^.

Life-history traits are often negatively correlated with each other because of life-history trade-offs, and these exchanges play important roles in insect development^10^. A trade-off between two traits occurs when an increase in fitness in one is followed by a decrease in fitness in the other, because of a concomitant change^11^. Trade-offs between reproductive investment and somatic maintenance, especially that between reproduction and lifespan or stress resistance under specific conditions, have been studied extensively^12^. A negative correlation between fecundity and longevity has been found throughout multicellular organisms owing to qualitative and quantitative dietary manipulations. These studies have gained considerable empirical support in many organisms, including *Anastrepha ludens*, *Trichopria drosophilae*, *Oncopeltus fasciatus* and *Drosophila melanogaster* ^13–16^. Phenotypic manipulations have also revealed trade-offs between reproduction and stress-related traits, including starvation, and thermal- and oxidative-stress resistance^17–19^. There are also trade-offs between desiccation and starvation resistance, heat- and cold-stress resistance, and longevity and starvation resistance^20–22^.

The ability to switch to new dietary sources can help insects survive in fluctuating environments, and the regulation of gene expression may be one way an organism can adapt. As different dietary sources vary in nutritional content, some genes associated with various biochemical pathways may be expressed differentially in response to different dietary states. Determining the underlying genetic and molecular mechanisms involved in adapting to different dietary states will help us to better understand the relationships among phenotype, gene expression and environment^23^. A number of genes related to metabolic function and stress have been identified by switching *D. melanogaster* from a cornmeal medium to pure bananas^24^. Moreover, in an immediate dietary switch, 144 “switching genes” associated with mRNA processing and protein translation pathways were identified and may be involved in mediating longevity in *D. melanogaster* ^25^.

The oriental fruit fly, *Bactrocera dorsalis* (Hendel), is a notorious pest of agricultural fruit across India, East Asia and the Pacific^26^. It attacks over 250 host plants, including many types of commercial fruit, such as carambola, peach, citrus and mango, a wide variety of other agricultural products, such as coffee and chilli pepper, and wild hosts^27^. A previous study found that the extension of lifespan by DR is evolutionarily conserved in *B. dorsalis* and that yeast: sugar ratios significantly modulate lifespan in this species^28^. However, little is known about the effects of DR and dynamic diets on the phenotypes (the responses of stress resistance and hatchability to DR; the responses of lifespan and fecundity to dynamic diets) and gene expressions in *B. dorsalis*. In addition, the *B. dorsalis* genome is well annotated (NCBI Assembly: ASM78921v2), which allows identification of genes and gene families that may be up- or down-regulated in response to different diets. Therefore, we investigated the effects of chronic DR and control diets (CD) on the plasticity of lifespan, fecundity, stress resistance (starvation, desiccation, heat and cold resistance), hatchability and gene expression in *B. dorsalis*. Moreover, we also examined lifespan, reproduction and gene expression in response to an acute dietary switch, CD to DR (CDS) or DR to CD (DRS), in *B. dorsalis*. The aim of this study was to facilitate a better understanding of the molecular mechanisms responsible for phenotypic adjustments in *B. dorsalis* to the nutritional stress and fluctuating dietary conditions.

## Results

### Starvation and desiccation resistances

**Figure 1:**
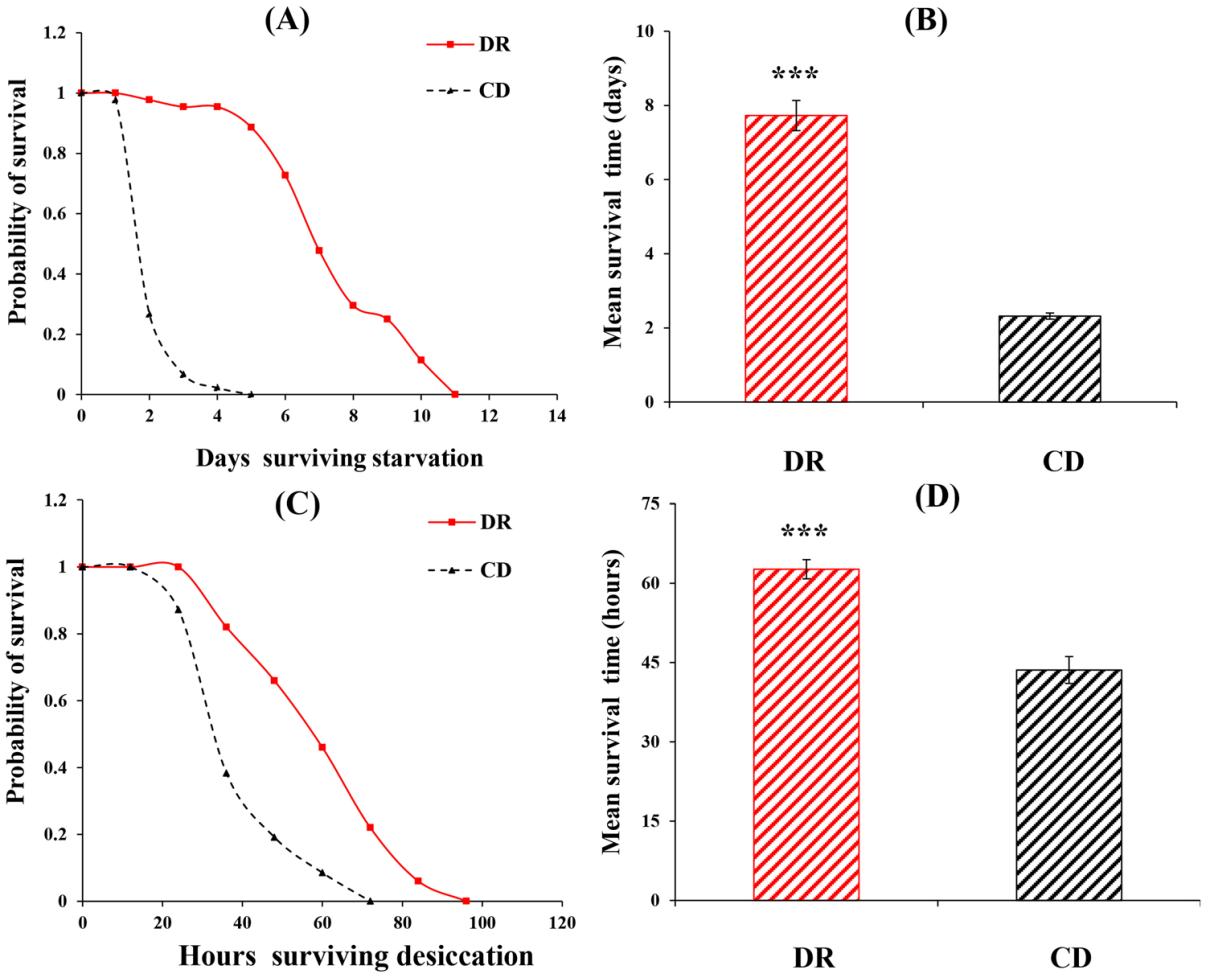
Survival curves (Each curve represents the cumulative survival probability for each diet treatment) for starvation resistance (**A**) and desiccation resistance (**C**), and mean time before death from starvation stress (**B**) and desiccation stress (**D**) in *Bactrocera dorsalis* derived from either dietary restriction (DR) or control diet (CD) regimes. Significant differences between the two treatments were analysed by the independent samples t-test (for comparison of two means) (*P < 0.05, **P < 0.01, ***P < 0.001).

For the starvation treatment, *B. dorsalis* developed on the DR treatment had longer survival times than flies developed on the CD (Kolmogorov-Smirnov test, n_DR_ = 42; n_CD_ = 35, Z = 3.13, *P* < 0.001) (Fig. 1A), with mean survival times of 7.73 ± 0.41 and 2.32 ± 0.08 days, respectively (*P* < 0.001) (Fig. 1B). For the desiccation treatment, DR could significantly increase the desiccation resistance (Kolmogorov-Smirnov test, n_DR_ = 44; n_CD_ = 33, Z = 2.06, *P* < 0.001) (Fig. 1C), and the mean survival times were 62.64 ± 1.80 and 43.57 ± 2.53 h under the DR and CD treatments, respectively (*P* < 0.001) (Fig. 1D).

### Heat and cold resistances

**Figure 2:**
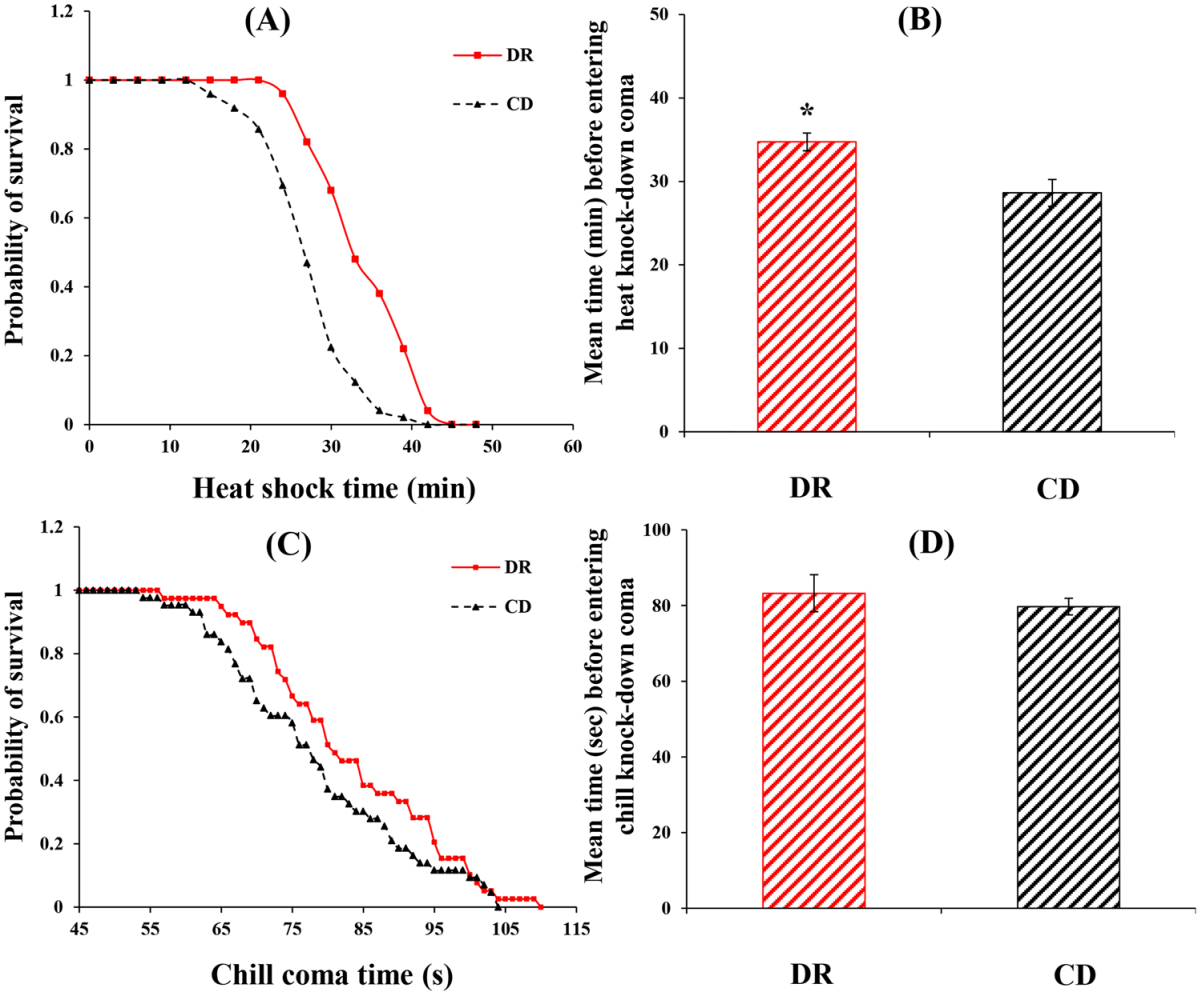
Survival curves (Each curve represents the cumulative survival probability for each diet treatment) for heat shock (**A**) and cold shock (**C**), and mean time entering heat shock (**B**) and cold shock (**D**) in *Bactrocera dorsalis* derived from either dietary restriction (DR) or control diet (CD) regimes. Significant differences between the two treatments were analysed by the independent samples t-test (for comparison of two means) (*P < 0.05, **P < 0.01, ***P < 0.001).

DR could significantly increase the heat resistance of *B. dorsalis* (Kolmogorov-Smirnov test, n_DR_ = 76; n_CD_ = 58, Z = 1.14, *P* = 0.036) (Fig. 2A). Flies on the DR treatment took longer (34.74 ± 1.06 min) to experience heat shock at 42 °C than flies on the CD (28.63 ± 1.61 min) (*P = *0.016) (Fig. 2B). However, there was no dietary influence on low-temperature sensitivity (Kolmogorov-Smirnov test, n_DR_ = 86; n_CD_ = 65, Z = 0.43, *P* = 0.99) (Fig. 2C). The time for *B. dorsalis* to experience cold shock at 5 °C was not significantly different between DR and the CD (*P = *0.50) (Fig. 2D).

### Hatchability

**Figure 3:**
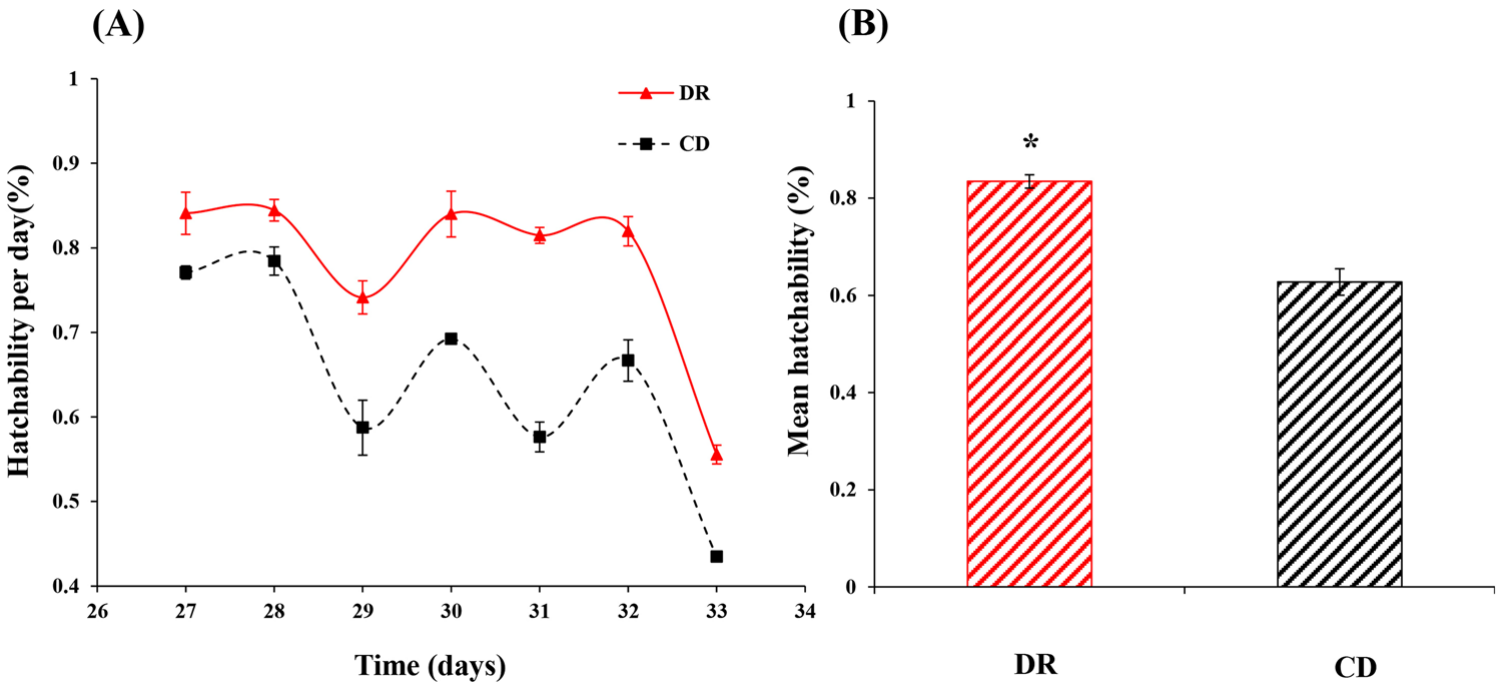
Daily hatching rate of eggs laid during a seven day period (**A**) and mean hatchability of all the eggs laid in 7 days (**B**) by *Bactrocera dorsalis* females derived from either dietary restriction (DR) or control diet (CD) regimes from 27 to 33 days after emergence. Each value represents the mean ± SE of three replicates. Significant differences between the two treatments were analysed by the independent samples t-test (for comparison of two means) (*P < 0.05, **P < 0.01, ***P < 0.001).

There was a significant dietary effect on the hatchability (Fig. 3A), and the mean hatching rates of the total eggs laid during a seven-day period (days from 27 to 33 after emergence) differed significantly between DR (83.45%) and the CD (62.74%) (*P = *0.048) (Fig. 3B). Flies developed on the DR treatment had higher hatching rates than flies developed on the CD, which suggested that DR could significantly increase the hatchability of *B. dorsalis*.

### Fecundity in response to dietary switches

**Figure 4:**
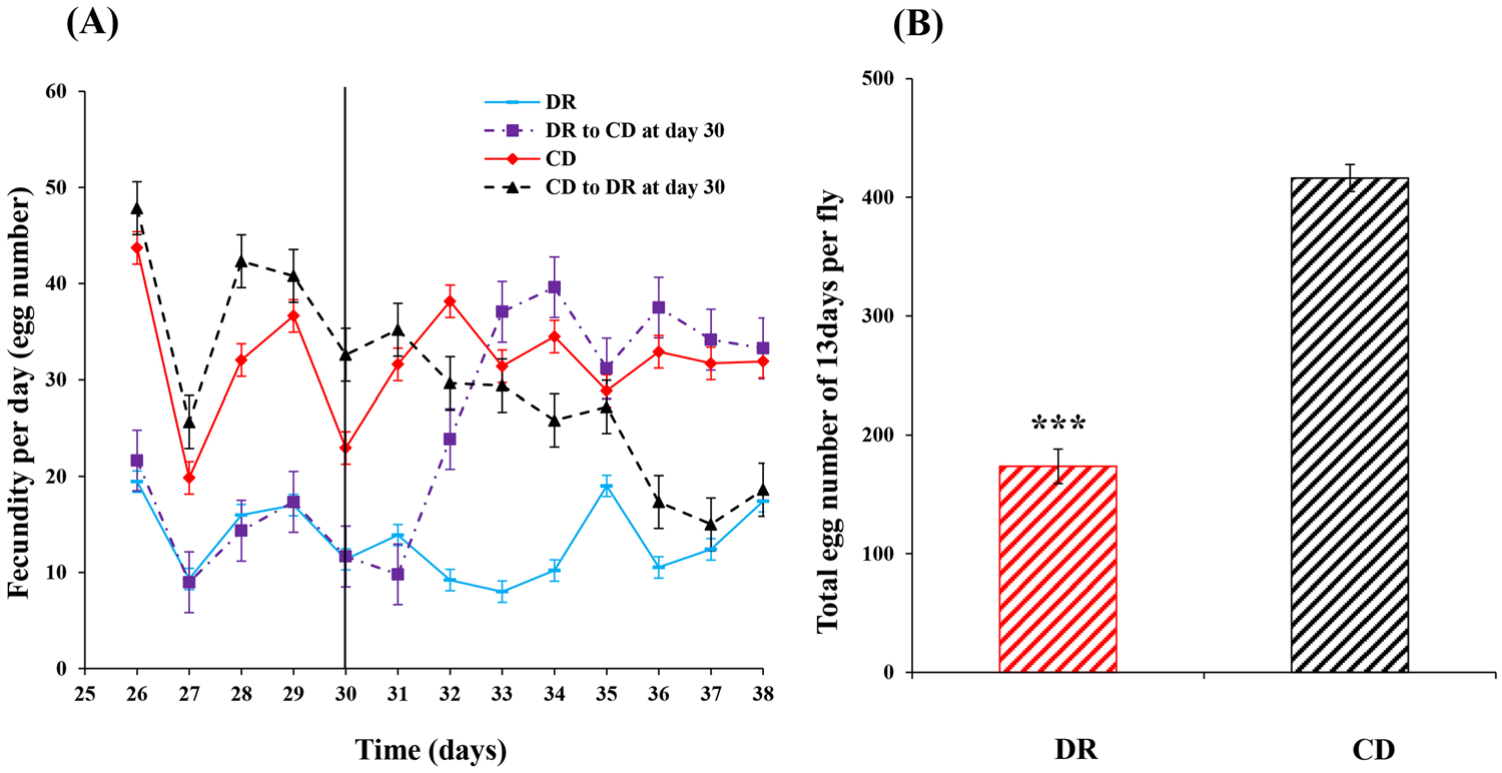
Daily number of eggs laid per female *Bactrocera dorsalis* upon dietary switch at day 30 after emergence. Each value represents the mean ± SE of five replicates (**A**). Mean number of eggs laid in 13 days, from 26 to 38 days after emergence, per female *Bactrocera dorsalis* under chronic dietary restriction (DR) and control diet (CD) regimes (**B**). Significant differences between the two treatments were analysed by the independent samples t-test (for comparison of two means) (*P < 0.05, **P < 0.01, ***P < 0.001).

There was a significant effect of diet regimes on fecundity (Fig. 4A), and females fed on the CD consistently laid significantly more eggs than those on the DR treatment (*P < *0.001) (Fig. 4B). When the CDS switch occurred, flies firstly experienced a 24-h adaptive phase, and then the fecundity gradually reduced. Eight days later, the fecundity dropped down to the DR regime level (Fig. 4A). When the DRS switch occurred, flies also firstly experienced a 24-h adaptive phase, but then the fecundity rapidly increased. Three days later, the fecundity ascended to the CD regime level (Fig. 4A).

### Age-specific survivorship in response to dietary switches

**Figure 5:**
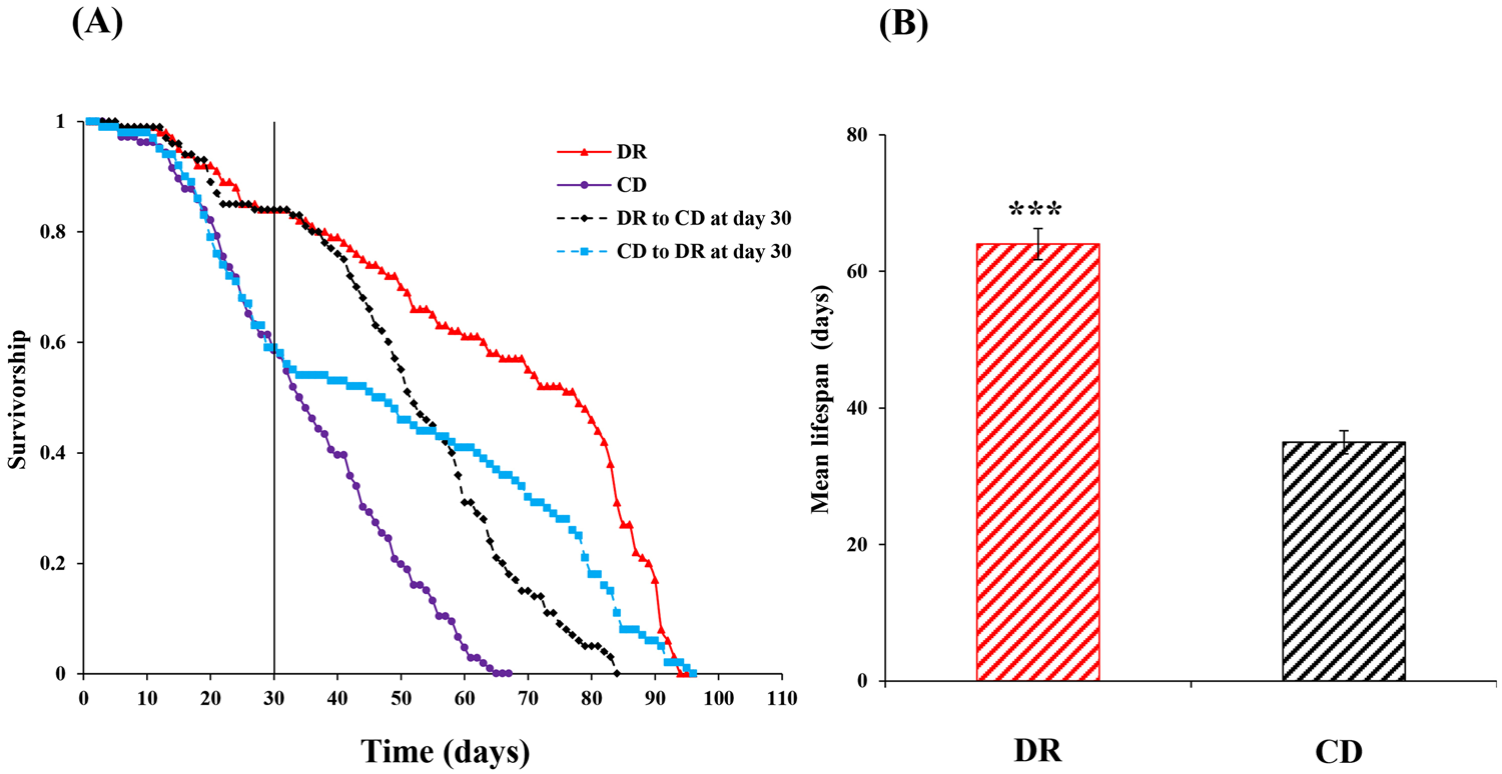
Survival curves (Each curve represents the cumulative survival probability for each diet treatment) for *Bactrocera dorsalis* adults upon dietary switch at day 30 after emergence (**A**). Mean lifespans of *Bactrocera dorsalis* adults fed on chronic dietary restriction (DR) and control diet (CD) (**B**). Significant differences between the two treatments were analysed by the independent samples t-test (for comparison of two means) (*P < 0.05, **P < 0.01, ***P < 0.001).

There was a significant effect of diet regimes on lifespan (Kolmogorov-Smirnov test, n_DR_ = 100; n_CD_ = 100, Z = 1.94, *P* < 0.001) (Fig. 5A), and flies fed on the DR treatment (63.38 ± 2.28 days) lived longer than that those on the CD (35.46 ± 1.70 days) (*P < *0.001) (Fig. 5B). The DRS switch in *B. dorsalis* caused the survivorship of the switched cohorts to significantly decrease, and they tended to adopt the survivorship level of the chronic CD cohorts. In contrast, the CDS switch caused the survivorship of the switched cohorts to significantly increase, and they tended to adopt the survivorship level of the chronic DR cohorts (Fig. 5A).

### Quality assessment of the sequencing data for each *B. dorsalis* sample

In this study, 12 samples of *B. dorsalis* were sequenced using RNA-Seq technology. RNASeq raw sequences generated using complete genomics have been submitted to SRA at NCBI under the Accession no. SAMN05284482. In total, 23,952,309 clean reads out of 23,957,385 raw reads were obtained for paired-end sequencing, and the average length of the sequence reads was 50 bp (see Supplementary Table S1). To evaluate whether the sequencing data were qualified, we conducted a strict quality control analysis of each *B. dorsalis* sample, and the average genome mapping ratio was 83.98% (see Supplementary Table S2).

### Effects of diets on gene expressions in *B. dorsalis*

In total, 230 genes were differentially expressed between the chronic DR and CD cohorts, and the magnitudes of these changes were defined as |log2(Y/X)| ≥ 1, with divergence probability ≥0.8. Of these genes, 177 (77%) with up-regulated expression levels and 53 (23%) with down-regulated expression levels (see Supplementary Figure S1A and Table S3). For the KEGG analysis (obtained by KEGG, http://www.kegg.jp/kegg/kegg1.html), the up-regulated pathways included ‘Phototransduction - fly’ (ko04745, 11.21%, *P* = 2.6e-13), ‘Tight junction’ (ko04530, 8.62%, *P* = 1.9e-05), ‘Calcium signalling pathway’ (ko04020, 7.76%, *P* = 2.7e-05), ‘Leukocyte transendothelial migration’ (ko04670, 8.6%, *P* = 2.7e-05), ‘Platelet activation’ (ko04670, 6.9%, *P* = 0.0026) and ‘Folate biosynthesis’ (ko00790, 1.72%, *P* = 0.042) (Table 1). Importantly, the oxidative phosphorylation pathway (ko00190, 6.03%, *P* = 0.0013) (obtained by KEGG, http://www.kegg.jp/kegg/kegg1.html) was also significantly enriched, and the energy production-related genes associated with ATP synthase were up-regulated (see Supplementary Figure S2). Down-regulated genes were particularly associated with ‘digestive system’ and ‘metabolism’ pathways (Table 2) (*P* < 0.05). Such as the expression levels of *vitellogenin-1* (*Vg-1*) (gene ID: 105232170), *vitellogenin-2* (*Vg-2*) (gene ID: 105222970) and *larval serum protein-2* (*Lsp-2*) (gene ID: 105225725) were down-regulated by 4.09-, 2.84- and 15.30-fold, respectively, under the chronic DR regime when compared with their expression levels under the chronic CD regime (see Supplementary Table S3).

**Table 1:** KEGG pathways significantly enriched for up-regulated genes in dietary restriction (DR) versus control diet (CD).

KEGG pathway	Pathway ID	DEGs (116)	*P*-value	Level 1	Level 2
Phototransduction - fly	ko04745	13 (11.21%)	2.6e-13	Organismal Systems	Sensory system
Cardiac muscle contraction	ko04260	9 (7.76%)	1.12e-08	Organismal Systems	Circulatory system
Oxytocin signaling pathway	ko04921	11 (9.48%)	9.9e-08	Organismal Systems	Endocrine system
Adrenergic signaling in cardiomyocytes	ko04261	9 (7.76%)	8.2e-06	Organismal Systems	Circulatory system
Tight junction	ko04530	10 (8.62%)	1.9e-05	Cellular Processes	Cellular community
Calcium signaling pathway	ko04020	9 (7.76%)	2.7e-05	Environmental Information Processing	Signal transduction
Focal adhesion	ko04510	12 (10.34%)	5.1e-05	Cellular Processes	Cellular community
Regulation of actin cytoskeleton	ko04810	12 (10.34%)	8.8e-05	Cellular Processes	Cell motility
Leukocyte transendothelial migration	ko04670	10 (8.62%)	0.00011	Organismal Systems	Immune system
Pancreatic secretion	ko04972	10 (8.62%)	0.00021	Organismal Systems	Digestive system
cGMP-PKG signaling pathway	ko04022	8 (6.91%)	0.00025	Environmental Information Processing	Signal transduction
Two-component system	ko02020	4 (3.45%)	0.00055	Environmental Information Processing	Signal transduction
Oxidative phosphorylation	ko00190	7 (6.03%)	0.0013	Metabolism	Energy metabolism
Renin secretion	ko04924	5 (4.31%)	0.0014	Organismal Systems	Endocrine system
Vascular smooth muscle contraction	ko04270	6 (5.17%)	0.0017	Organismal Systems	Circulatory system
Gastric acid secretion	ko04971	5 (4.31%)	0.0018	Organismal Systems	Digestive system
Phototransduction	ko04744	3 (2.59%)	0.0020	Organismal Systems	Sensory system
Rap1 signaling pathway	ko04015	10 (8.62%)	0.0023	Environmental Information Processing	Signal transduction
Platelet activation	ko04611	8 (6.9%)	0.0026	Organismal Systems	Immune system
Circadian entrainment	ko04713	5 (4.31%)	0.0034	Organismal Systems	Environmental adaptation
Adherens junction	ko04520	6 (5.17%)	0.0036	Cellular Processes	Cellular community
Plant-pathogen interaction	ko04626	3 (2.59%)	0.0039	Organismal Systems	Environmental adaptation
Aldosterone synthesis and secretion	ko04925	5 (4.31%)	0.0052	Organismal Systems	Endocrine system
Hippo signaling pathway	ko04390	6 (5.17%)	0.0061	Environmental Information Processing	Signal transduction
Olfactory transduction	ko04740	4 (3.45%)	0.0065	Organismal Systems	Sensory system
Carbon fixation pathways in prokaryotes	ko00720	2 (1.72%)	0.014	Metabolism	Energy metabolism
Glyoxylate and dicarboxylate metabolism	ko00630	3 (2.59%)	0.014	Metabolism	Carbohydrate metabolism
Citrate cycle (TCA cycle)	ko00020	3 (2.59%)	0.017	Metabolism	Carbohydrate metabolism
cAMP signaling pathway	ko04024	6 (5.17%)	0.017	Environmental Information Processing	Signal transduction
Estrogen signaling pathway	ko04915	4 (3.45%)	0.023	Organismal Systems	Endocrine system
Protein digestion and absorption	ko04974	6 (5.17%)	0.031	Organismal Systems	Digestive system
Salivary secretion	ko04970	4 (3.45%)	0.035	Organismal Systems	Digestive system
Hippo signaling pathway - fly	ko04391	4 (3.45%)	0.036	Environmental Information Processing	Signal transduction
Neuroactive ligand-receptor interaction	ko04080	7 (6.03%)	0.037	Environmental Information Processing	Signaling molecules and interaction
Dopaminergic synapse	ko04728	4 (3.45%)	0.042	Organismal Systems	Nervous system
Folate biosynthesis	ko00790	2 (1.72%)	0.042	Metabolism	Metabolism of cofactors and vitamins
Long-term potentiation	ko04720	3 (2.59%)	0.045	Organismal Systems	Nervous system

The Bonferroni Correction method was used for the Multiple Hypothesis Test correction, and False Discovery Rate-corrected *P* < 0.05 was the cut-off.

**Table 2:** KEGG pathways significantly enriched for down-regulated genes in dietary restriction (DR) versus control diet (CD).

KEGG pathway	Pathway ID	DEGs (19)	*P*-value	Level 1	Level 2
Protein digestion and absorption	ko04974	4 (21.05%)	0.00051	Organismal Systems	Digestive system
Pancreatic secretion	ko04972	4 (21.05%)	0.00067	Organismal Systems	Digestive system
Neuroactive ligand-receptor interaction	ko04080	4 (21.05%)	0.0014	Environmental Information Processing	Signaling molecules and interaction
PPAR signaling pathway	ko03320	2 (10.53%)	0.015	Organismal Systems	Endocrine system
Glycerolipid metabolism	ko00561	2 (10.53%)	0.021	Metabolism	Lipid metabolism
Riboflavin metabolism	ko00740	1 (5.26%)	0.029	Metabolism	Metabolism of cofactors and vitamins
Betalain biosynthesis	ko00965	1 (5.26%)	0.033	Metabolism	Biosynthesis of other secondary metabolites
Isoquinoline alkaloid biosynthesis	ko00950	1 (5.26%)	0.031	Metabolism	Biosynthesis of other secondary metabolites
Glycosaminoglycan degradation	ko00531	1 (5.26%)	0.033	Metabolism	Glycan biosynthesis and metabolism
Glyoxylate and dicarboxylate metabolism	ko00630	1 (5.26%)	0.039	Metabolism	Carbohydrate metabolism
Terpenoid backbone biosynthesis	ko00900	1 (5.26%)	0.045	Metabolism	Metabolism of terpenoids and polyketides
Insect hormone biosynthesis	ko00981	1 (5.26%)	0.047	Metabolism	Metabolism of terpenoids and polyketides

The Bonferroni Correction method was used for the Multiple Hypothesis Test correction, and False Discovery Rate-corrected *P* < 0.05 was the cut-off.

Of the 31 DEGs across the CDS and CD treatments that had greater than 2-fold changes, 24 transcripts (77.4%) showed down-regulated expression and 7 transcripts (22.6%) showed up-regulated expression (see Supplementary Figure S1B and Table S4). For the KEGG analysis, the majority of down-regulated pathways belonged to the ‘digestive system’ and ‘metabolism’. For instance, ‘Pancreatic secretion’ (ko04972, 38.46%, *P* = 0.0013), ‘Protein digestion and absorption’ (ko04974, 21.05%, *P* = 0.0001), ‘Drug metabolism - other enzymes’ (ko00983, 15.38%, *P* = 0.0066), ‘Glycerophospholipid metabolism’ (ko00564, 15.38%, *P* = 0.0066), ‘Fructose and mannose metabolism’ (ko00051, 7.69%, *P* = 0.039) and ‘Tyrosine metabolism’ (ko00350, 7.69%, *P* = 0.049). We also found the up-regulated genes were significantly enriched in three KEGG pathways including ‘Protein digestion and absorption’ (ko04974, 33.33%, *P* = 0.0059), ‘Pancreatic secretion’ (ko04972, 33.33%, *P* = 0.0067), ‘Neuroactive ligand-receptor interaction’ (ko04080, 33.33%, *P* = 0.01) (see Supplementary Table S5), Furthermore, 58 genes showed significant changes between the DRS and DR regimes, with 31 up-regulated genes (53.4%) and 27 down-regulated genes (46.6%) having greater than 2-fold changes (see Supplementary Figure S1C and Table S6). For the KEGG enrichments, the up-regulated genes were particular associated with ‘metabolism’ pathways, including ‘Riboflavin metabolism’ (ko00740, 7.69%, *P* = 0.00017), ‘Betalain biosynthesis’ (ko00965, 15.38%, *P* = 0.00023), ‘Glycerolipid metabolism’ (ko00561, 23.08%, *P* = 0.00044) and ‘Tyrosine metabolism’ (ko00350, 15.38%, *P* = 0.0037). The down-regulated genes were significantly enriched in two immune pathways including ‘Leukocyte transendothelial migration’ (ko04670, 33.33%, *P* = 0.0058), and ‘Platelet activation’ (ko04611, 33.33%, *P* = 0.006) (see Supplementary Table S7). Remarkably, the expression of *Vg-1* (gene ID: 105232170), *Vg-2* (gene ID: 105222970), *Lsp-1* (gene ID: 105227437), and *Lsp-2* (gene ID: 105225725) showed 9.01-, 4.26-, 12.21- and 36.00-fold increases under the DRS treatment when compared with the DR treatment (see Supplementary Table S6).

## Discussion

In this study, we found that the stress resistance and lifespan of *B. dorsalis* significantly increased under the DR regime with a reduced fecundity. The current study strongly suggested a trade-off between reproduction and stress resistance, as well as lifespan, depending on the quality of the available diet, which confirmed the theory of energy or resource allocation trade-offs in *B. dorsalis*.

*B. dorsalis* that developed on the DR treatment had a higher starvation resistance level than flies developed on the CD. The increase in the lipid content and energy metabolites may be the underlying reasons, and producing high reserves of lipids for starvation resistance is likely to be costly^29^. Thus, a trade-off may exist with the life-history traits involving a resource allocated either to stress resistance or to ovarian activity^20^. In addition, *D. melanogaster* have increased levels of starvation resistance was also more resistant to desiccation^30^. Here, we also found that desiccation resistance had a positive correlation with starvation resistance in *B. dorsalis* under the DR regime. The higher desiccation resistance may result from the metabolic end products of proteins, such as uric acid, which had a protective effect against the increasing osmotic pressure during desiccation by reducing the water loss from cells^31^. *B. dorsalis* that developed on the DR treatment had a higher heat resistance than flies fed on the CD, which corroborated findings that DR or mild starvation can increase longevity, as well as resistance to stressors, such as heat stress^32^. However, *Drosophila* that developed on protein-enriched media have higher heat resistance levels than flies grown on carbohydrate-enriched media, which demonstrated the complexity of organismal nutrient acquisition and utilization^5^. Here, there was no difference in the cold resistance between DR and CD regimes. The result was consistent with a previous study in which DR initially had no effect on cold-stress resistance^33^. Furthermore, longevity is causally related to the ability to withstand extrinsic or intrinsic stresses, and extended longevity is often accompanied with increased resistance against various stressors^34^. In studies using artificial selection regimes, genetic correlations between resistance to several stresses and longevity were found, which strongly suggested an overlap in the genetic basis for lifespan and stress resistance^35^. Interestingly, our current results confirmed the previous study that DR extended lifespan in *B. dorsalis* ^28^. A positive correlation between lifespan and stress resistance was found in this study, which showed that the longer lifespan was accompanied by higher stress resistance under the DR regime in *B. dorsalis*. Additionally, the dietary quality could affect hatching success, and even with less egg production, DR significantly increased the hatching rate when compared with the CD regime. An alternative explanation may be that many of the eggs produced on the CD treatment were resting eggs^36^. In addition, total egg energy may be related to hatching success^37^. Hence, we inferred that to complete fly population development, *B. dorsalis* adopted a strategy that decreased the egg production and increased energy investment per egg at the same time to combat the chronic nutritional stress conditions.

In the present study, we also investigated the dynamic changes of fecundity and lifespan in response to the dietary switch in *B. dorsalis*. The results showed that fecundity had a negative relationship with lifespan upon dietary switches as well. When the diet was switched from the CD to DR, *B. dorsalis* might reduce reproductive effort, thereby increased the allocation of resources to somatic maintenance and survival, until the nutritional conditions improved (diet was switched from DR to CD), the fecundity of *B. dorsalis* could resume with the lifespan decreased to the normal level. The results suggested that there was a trade-off between fecundity and longevity in response to dietary switches, and the acute DR may also trigger an adaptive reallocation of resources between reproduction and somatic maintenance^13^. It was worth noting that *B. dorsalis* adopt the more fecundity strategy upon the dietary switch, and the fecundity of flies responded more sensitive to DRS than CDS. Namely, when the diet was switched from DR to CD, *B. dorsalis* increased their fecundity quickly, and *vitellogenin-1* and *vitellogenin-2* were also up-regulated within 24 h. On the contrary, when the diet was switched from CD to DR, *B. dorsalis* decreased their fecundity slowly, and the expression of *vitellogenin* showed no dynamic changes within 24 h. In addition, upon the acute dietary switches, the DEGs were particularly associated with metabolism and digestive pathways, which suggest that metabolism and digestive activity might be involved in the ability of *B. dorsalis* to utilize dynamic dietary resources^24^. However, we also found when the flies were switched from DR to CD, the genes involved in metabolism were affected but the genes involved in digestion pathway were not affected within 24 h after the dietary switch. The explanation may be that the flies rapidly increase their *vitellogenin* expression by metabolism of reserved amino acid and relocate more energy to reproductive investment, which may explain that the number of fecundity of flies quickly reached to the level of CD levels within three days after the dietary switch. Secondly, we speculated that the genes involved in digestion might be affected in response to the differences in protein:carbohydrate ratio after 24 h (not within 24 h) when the dietary switch occurred.

Here, we also found *B. dorsalis* could mount a strong transcriptional response to the different diet environments. Upon the acute dietary switch (CDS and DRS regimes), DEGs were particularly associated with metabolism and digestive pathways, suggesting that *B. dorsalis* can facilitate switches between food sources through differential expression of genes related to metabolism and digestive activities, which may be involved in the ability of *B. dorsalis* to use fluctuating dietary resources^24^. The different patterns of gene regulation exhibited in response to the diets may reflect differences in macromolecular content of these two food sources. In particular, DR contains relatively less proteins, and relatively fewer lipids, than CD. The down-regulation of genes related to protein digestion and absorption, and lipid metabolism under DR treatment is consistent with these differences. Previous studies have shown that rates of lipid and protein metabolism change in various *Drosophila* species under stressful conditions found in natural settings, such as low food availability^38^. It has been reported that immune system was affected by the macronutrient content of the diet, and when given a choice between diets that differ in their macronutrient composition, pathogen-infected individuals would select a diet that improves their survival, suggesting that the nutritional composition of the diet could play a role in defense against disease^39^. Moreover, the previous studies found that dietary restriction could increase the immune traits and resistance to parasitism^40, 41^. Similarly, we found that DR could improve their immune response (up-regulated genes involved in immunity), suggesting that DR play a role in defense against disease and improves the survival of *B. dorsalis*. The down-regulation of genes involved in insect immunity when DRS (switch from DR to CD) was significantly enriched in two immune pathways compared to DR treatment. The explanation might be that DRS could modify their allocation of nutrients to improve their reproduction as well as decrease their immune traits and survival within 24 h after dietary switch in *B. dorsalis*. DR also up-regulated the calcium-signalling pathway, and calcium and calmodulin are the key components of signal transduction pathways, which could be involved in responses to various environmental stress conditions^42^. Lsp-2 is involved in nutrient reservoir activities, synaptic target inhibition and motor neuron axon guidance^43^. Here, *Lsp-2* was down-regulated under the DR treatment, and *Lsp-2* was also categorized as switching genes under CDS and DRS treatments. In *D. melanogaster*, *Lsp-2* was the only gene down-regulated among three related longevity-inducing interventions, DR, dSir2 over expression and DN-Dmp53 expression in long-lived flies, which strongly suggested that *Lsp-2* may play an important role in regulating the lifespan of *B. dorsalis* under chronic DR regimes^44^.

Additionally, DR activated the energy metabolism pathways, including oxidative phosphorylation pathway and arbon fixation pathways in prokaryotes. These two pathways play a central role in eukaryotic metabolism to provide the energy, in the form of ATP, required for survival in insects^45^. The overexpression of energy production-related genes in *B. dorsalis* under chronic DR treatments may reflect the costs necessary for surviving under those conditions. Therefore, we hypothesized that chronic DR enhanced the energy metabolism pathways, which increased energy production to adapt and refill the energy deficiency caused by long-term DR in *B. dorsalis* ^46^. Additional, *Vg-1* and *Vg-2* genes that enriched in metabolism pathway are down-regulated, which may lead to the decreased fecundity of *B. dorsalis* under DR regimes in our study^47^. The up-regulated genes involved in energy production and the down-regulated metabolism pathway involved in egg production may mediate a trade-off between somatic maintenance and reproduction under DR treatment^48^.

## Conclusion

Our study documented many remarkable differences in stress resistance, life-history traits and gene expression associated with different dietary states, which suggested the *B. dorsalis* undergoes phenotypic plasticity, life-history trade-offs, and gene expression changes in response to its dietary environment. These datasets also raise questions about the role of diets, and specifically the dietary protein:carbohydrate ratio, in maintaining trait variation within and among populations. The ability to use different food sources is likely under strong selection when *B. dorsalis* is faced with natural variations in macro-nutrient (protein, carbohydrate and lipid) availability. A proteomics analysis would be useful to investigate the changes at the transcriptome level as reflected in the protein mechanisms and to confirm the relationship between genotype and phenotype under different dietary conditions.

## Methods

### Insects

The laboratory colony of *B. dorsalis* was originally collected from Dongguan in Guangdong Province, China, in 2008. The insects were reared in plastic cages at 27 ± 1 °C, 70 ± 5% relative humidity, and a photoperiod of 14 h:10 h (L:D). The larvae were fed on an artificial diet consisting of yeast powder, sucrose and corn and wheat flour. The third-instar larvae were transferred into a plastic basin containing sand for pupation. Pupae were sieved from the sand and placed in plastic cages with adult food (sucrose: yeast hydrolysate = 3:1) and water. *B. dorsalis* were all fed on the control diets (CD) before the current experiments were performed.

### Experimental diets

The experimental diets for adult flies used in this study were all based on a previous study on fruit flies^7^. We varied the amount of yeast extract (Oxoid Ltd., Basingstoke, Hampshire, England) per 100 g of adult diet to a yeast: sucrose ratio of 4.76:95.24 (4.76% yeast) for the dietary restriction (DR) and 25: 75 (25% yeast) for the CD. The yeast was comprised of 62.5% protein, and also contained the water-soluble B-complex vitamins, sodium chloride with pH 7.0 ± 0.2 (0.5% solution) at 25 °C, and the yeast: sucrose ratios resulted in protein:carbohydrate ratios under DR and in the CD of 2.98:95.24 and 15.63:75.00, respectively. Strictly speaking the diets in these experiments varied both the yeast and the sucrose contents. Because the *B. dorsalis* were given *ad libitum* access to the diets, we assumed that the sucrose in each of the diets is not limited, and that any response to DR is mediated primarily through the variation in the yeast content.

The life-history traits, dietary switches, and gene expressions were measured at the time that there was a measurable separation between the mortality trajectories of the CD and DR cohorts, according to the previous studies^49^, In this study, we found a consistent 2-fold difference in age-specific instantaneous mortality between cohorts at day 30 after emergence. Therefore, the life-history traits were tested and the dietary switches also occurred at day 30 after emergence of *B. dorsalis*.

### Starvation and desiccation treatment

For the starvation treatment, the female flies (n = 50 females) from CD and DR treatments at day 30 after emergence were transferred into new jars (five jars per treatment) containing small vessels plugged with cotton to prevent desiccation. After the flies were originally transferred, dead flies were recorded and removed daily until all of the flies had died. For the desiccation treatment, the female flies from each jar at day 30 after emergence were transferred into a new jar containing a disc of dry filter paper without adult diet. The observations were made as desiccation treatment.

### Heat and cold treatments

For the heat treatment, the female flies (n = 100 females) from each treatment at day 30 after emergence were transferred into new jars (10 jars per treatment). Then, the jars were hardened at 42 °C and evenly spaced on racks in incubators (Sanyo Electric Co., Ltd., Moriguchi-shi, Japan). The flies were scored as heat shocked when they dropped to the bottom of the jar and no longer moved. After the flies were originally transferred, we recorded the number of the heat shocked flies every 3 min. For the cold treatment, the treatment temperature was switched to 5 °C. After the flies were originally transferred, we recorded the times when flies appeared cold shocked.

### Egg hatching rate

Three hatchability replicates were conducted on each diet (n = 30 females per treatment). Females from each jar were allowed to oviposit into 2-mL microfuge tubes that were replaced daily. Eggs were collected daily during a seven day period (days 27–33 after emergence) from each treatment daily. Fifty eggs were selected randomly, and then transferred onto a fresh larval diet. The number of hatched larvae in the larval diet was counted to determine hatchability.

### Fecundity and age specific survivorship response to dietary switch

For CD, DR, CDS (acute dietary switch from CD to DR) and DRS (acute dietary switch from DR to CD) treatments, 5 jars containing 20 females and 20 males, resulting in a total of 20 jars (n = 100 females), were used to investigate the fecundity response. Females were allowed to oviposit into 2-mL microfuge tubes that were replaced daily. Eggs laid within a 24-h period were counted from 26 to 38 days, and the dietary shift was carried out at day 30 after emergence.

For each jar, 10 focal individuals of one sex were placed with 10 individuals of the opposite sex in each replicate (100 flies per treatment) to study the age specific survivorship response. Dead flies were recorded and removed daily, and the dietary switch was carried out at day 30 after emergence. Flies were maintained at a 1:1 sex ratio. If a focal individual in one of the mated treatments died, an opposite-sex individual was removed, and if a non-focal individual died, it was replaced with an age-matched individual of the same sex.

### Insect sample and RNA extraction

*B. dorsalis* were reared at a density of 15 males and 15 females per jar after emergence, and randomly assigned to the CD, DR, CDS (acute dietary switch from CD to DR) and DRS (acute dietary switch from DR to CD) treatments. The dietary switch occurred at day 30 after *B. dorsalis* emergence. Gene expressions were observed within 24 h following a dietary shift as observed in *D. melanogaster* ^22^. Here, we also found that *B. dorsalis* experienced a 24-h adaptive phase during the dietary switch. Therefore, we tested the gene expression of CDS and DRS at 24 h after the dietary shift, and female flies from each treatment were collected at 24 h after the diet switch for RNA extraction. Three replicates were performed for each treatment. RNA was extracted using the RNeasy plus Micro Kit (Qiagen GmbH, Hilden, Germany) following the manufacturer’s instructions, and the replicates for RNA extraction were from pooled adult females flies. RNA was quantified by measuring the absorbance at 260 nm using a NanoVue UV-Vis spectrophotometer (GE Healthcare Bio-Science, Uppsala, Sweden). The purities of the RNA samples were assessed at absorbance ratios of OD_260/280_ and OD_260/230_, and the RNA integrity was confirmed by 1% agarose gel electrophoresis.

### cDNA library construction and sequencing

Briefly, oligo(dT) magnetic beads were used to select mRNAs with polyA tails, and then the target RNA was obtained after purification. The target mRNAs were broken into short fragments by the addition of fragmentation buffer. The first-strand cDNA was generated using random hexamer-primed reverse transcription and was followed by the synthesis of the second-strand cDNA using RNase H and DNA polymerase I. The cDNA fragments were purified, and then were washed with EB buffer (10 mM Tris-Cl, pH 8.5) for end reparation, polyA addition and ligation to sequencing adapters. Two specific primers were used to amplify the ligation product. The PCR product was denatured by heat, and the single-stranded DNA was cyclized using a splint oligo and DNA ligase. The cDNA library was sequenced on a next-generation sequencing platform (BGI-seq500) using paired-end technology in a single run.

### Analysis of RNA-seq data

The raw reads produced by the complete genomics in this study were cleaned by discarding reads with adapters and reads in which there were more than 10% unknown bases. Low-quality reads, which have more than 50% low-quality bases, were removed as well. HISAT^50^ was used to map clean reads to the *B. dorsalis* reference genome, and Bowtie2^51^ aligned the reads to gene references using the default parameters. Gene expression levels in terms of transcripts were quantified using the RNA-Seq by expectation maximization and fragment per kilobase of exon model per million mapped reads method^52^.

The NOISeq method can screen differentially expressed genes (DEGs) between two groups, and maintains good true positive and false positive rates when increasing the sequencing depth^53^. Additionally, NOISeq models the noise distribution from the actual data. It can thus better adapt to the size of the dataset, and is more effective in controlling the rate of false discoveries. In this study, DEGs were determined using the NOISeq-bio methods. The fold changes (log_2_ ratio) were estimated according to the normalized gene expression level in each sample. We used the absolute value of log_2_ ratio >1 and probability >0.8 as the threshold to judge significant differences in gene expression^54^.

Pathway-based analyses helped to further understand gene biological functions, and the Kyoto Encyclopedia of Genes and Genomes (KEGG, the major public pathway-related database, http://www.kegg.jp/kegg/kegg1.html) was used to perform a pathway enrichment analysis of the DEGs^55–57^. A strict algorithm for the analysis, and the method used is described as follows:

$$P=1-\sum _{i=0}^{m-1}\frac{(\begin{array}{c}M\\ i\end{array})(\begin{array}{c}N-M\\ n-i\end{array})}{(\begin{array}{c}N\\ n\end{array})}$$

where *N* is the number of all of the genes having KEGG annotations; *n* is the number of DEGs in *N*; *M* is the number of genes that are annotated to specific pathways; and *m* is the number of DEGs in *M*. The calculated *P* value underwent a Bonferroni Correction, and the corrected *P* value ≤0.05 is the threshold. KEGG annotations fulfilling this condition are defined as KEGG pathways significantly enriched in DEGs.

### Statistical methods

The data of stress resistance, lifespan, fecundity and hatchability were analysed using SPSS 16.0 software (SPSS Inc., Chicago, IL, USA) and presented as mean ± standard error (SE). Significant differences between two treatments were analysed by the independent samples t-test (for comparison of two means) (*P < 0.05, **P < 0.01 and ***P < 0.001. The Kolmogorov-Smirnov test was used to compare the probability distributions of temporal curves in Figs 1, 2 and 5.

## Electronic supplementary material

Supplementary figures

Supplementary dataset
