# Supplementary material for: Phenotypic plasticity, trade-offs and gene expression changes accompanying dietary restriction and switches in Bactrocera dorsalis (Hendel) (Diptera: Tephritidae)
Source: Sci Rep. 2017 May 16;7:1988. doi: 10.1038/s41598-017-02106-3 (PMC5434071; doi:10.1038/s41598-017-02106-3)
Supplement: Supplementary file 1 — Supplementary figures [file 41598_2017_2106_MOESM1_ESM.pdf]

Supplementary Section

Phenotypic plasticity, trade-offs and gene expression changes accompanying dietary restriction and switches in *Bactrocera dorsalis* (Hendel) (Diptera: Tephritidae)

Er-Hu Chen, Qiu-Li Hou, Dan-Dan Wei, Hong-Bo Jiang and Jin-Jun Wang*

Key Laboratory of Entomology and Pest Control Engineering, College of Plant Protection, Southwest University, Chongqing 400715, People's Republic of China

Correspondence: Dr. Jin-Jun Wang, College of Plant Protection, Southwest University, Chongqing 400715, P. R. China. E-mail: wangjinjun@swu.edu.cn; jjwang7008@yahoo.com Tel: (86)-23-68250255; Fax: (86)-23-68251269

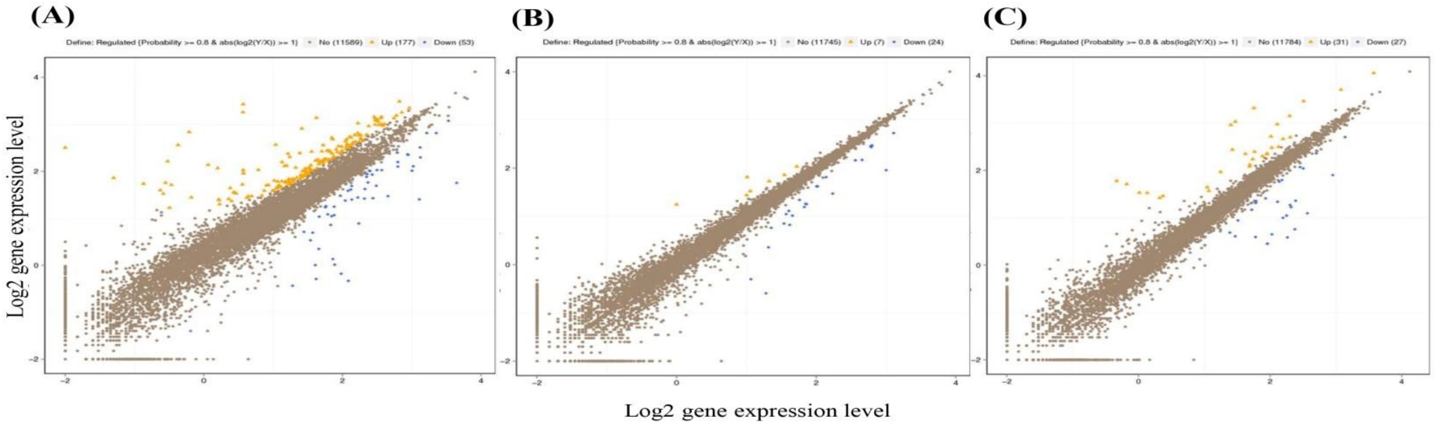

Figure S1. The number of differentially expressed genes (DEGs) between the control diet (CD; *x*-axis) and dietary restriction (DR; *y*-axis) treatments (A). Number of DEGs between CD (*x*-axis) and CD to DR switch (*y*-axis) treatments (B). Number of DEGs between DR (*x*-axis) and DR to CD switch (*y*-axis) treatments (C).The *x*-axis and *y*-axis present log2 values of gene expressions. Blue indicates down-regulated genes, orange indicates up-regulated genes and brown indicates non-regulated genes. The screening threshold is presented in the upper legend.

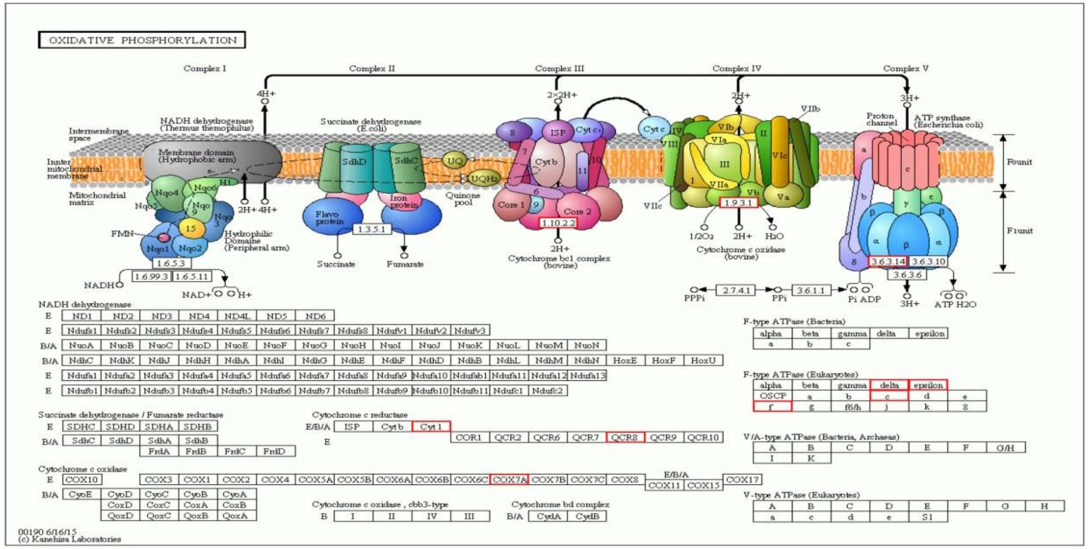

oxidative phosphorylation:

Gene ID：

105229356, 105223190, 105233695, 105233525, 105232504, 105223048, 105228336

Figure S2. The oxidative phosphorylation pathway (this image was obtained by Kyoto Encyclopedia of Genes and Genomes with permission from Kanehisa Laboratories) significantly responds to the chronic dietary restriction (DR) regime. Genes highlighted in red are enriched and up-regulated under the DR treatment.
